# A Decentralized Privacy-Preserving Healthcare Blockchain for IoT

**DOI:** 10.3390/s19020326

**Published:** 2019-01-15

**Authors:** Ashutosh Dhar Dwivedi, Gautam Srivastava, Shalini Dhar, Rajani Singh

**Affiliations:** 1 Institute of Computer Science, Polish Academy of Sciences, 01-248 Warsaw, Poland; ashudhar7@gmail.com (A.D.D.); rajanibabu7@gmail.com (R.S.); 2 Department of Mathematics and Computer Science, Brandon University, Brandon, MB R7A 6A9, Canada; 3 Research Center for Interneural Computing, China Medical University, Taichung 40402, Taiwan; 4 Department of Electronics and Communication, University of Allahabad, Allahabad 211002, India; dhar.shalini@rediffmail.com; 5 Faculty of Mathematics, Informatics and Mechanics, University of Warsaw, 02-097 Warsaw, Poland

**Keywords:** blockchain, medical big data, Internet of Things, smart contract, Ethereum, data preservation, key management, authentication, ring signature, smart cities

## Abstract

Medical care has become one of the most indispensable parts of human lives, leading to a dramatic increase in medical big data. To streamline the diagnosis and treatment process, healthcare professionals are now adopting Internet of Things (IoT)-based wearable technology. Recent years have witnessed billions of sensors, devices, and vehicles being connected through the Internet. One such technology—remote patient monitoring—is common nowadays for the treatment and care of patients. However, these technologies also pose grave privacy risks and security concerns about the data transfer and the logging of data transactions. These security and privacy problems of medical data could result from a delay in treatment progress, even endangering the patient’s life. We propose the use of a blockchain to provide secure management and analysis of healthcare big data. However, blockchains are computationally expensive, demand high bandwidth and extra computational power, and are therefore not completely suitable for most resource-constrained IoT devices meant for smart cities. In this work, we try to resolve the above-mentioned issues of using blockchain with IoT devices. We propose a novel framework of modified blockchain models suitable for IoT devices that rely on their distributed nature and other additional privacy and security properties of the network. These additional privacy and security properties in our model are based on advanced cryptographic primitives. The solutions given here make IoT application data and transactions more secure and anonymous over a blockchain-based network.

## 1. Introduction

**Figure 1:**
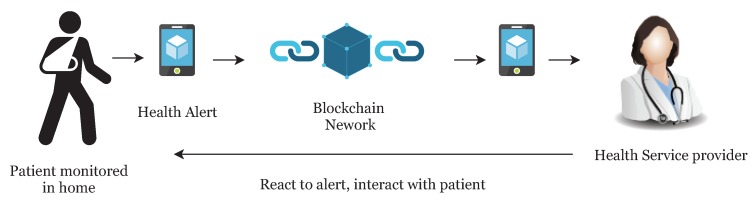
Remote Patient Monitoring.

Many countries are suffering from a dramatic increase in the number of medical patients, and it is becoming more difficult for patients to access primary doctors or caregivers. In recent years, the rise of IoT and wearable devices has improved the patient quality of care by remote patient monitoring. It also allows physicians to treat more patients. Remote patient monitoring (RPM) provides monitoring and care of patients outside of the conventional clinical setting (in the home as an example). Firstly, it allows patients an intrinsic convenience of service. Patients can stay connected with health providers as required. It also reduces medical costs and improves the quality of care. This is the main reason that healthcare providers are exploring means by which to provide RPM to the masses. The main component of a RPM system could be, a specially designed monitoring device to monitor and transmit health data to smart contracts, a smartphone with internet connectivity and an RPM application (Figure 1). Wearable devices and IoT play an important role in RPM and in the current push to develop Smart Cities. Wearable devices collect patient health data and transfer it to hospitals or medical institutions to facilitate health monitoring, disease diagnosis, and treatment. In doing so, we see a Big Data situation develop through all the patient data being analyzed and transferred.

**Figure 2:**
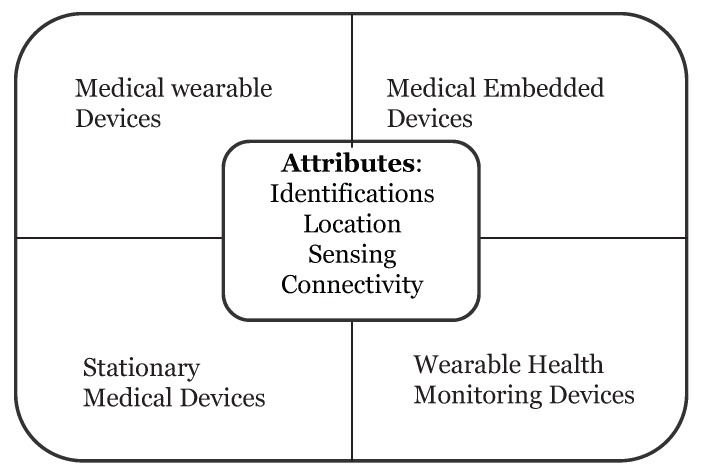
Healthcare IOT Topology.

Wearable devices in healthcare are smart electronic devices with micro-controllers that can be embedded into clothing or worn on the body as accessories. They are unobtrusive, user-friendly, and connected with advanced features such as wireless data transmission, real-time feedback and alerting mechanisms built into the device. These devices can provide important information to healthcare providers such as blood pressure, blood glucose levels and breathing patterns just to name a few. Healthcare devices can be categorized into four types (Figure 2):

- Stationary Medical Devices—devices can be used on a specific physical location (e.g., chemotherapy dispensing stations for home-based healthcare)
- Medical Embedded Devices—devices which can be implanted inside the body (e.g., pacemakers)
- Medical Wearable Devices—prescribed devices by doctors (e.g., insulin pump)
- Wearable Health Monitoring Devices—consumer products (e.g., Fitbit, Fuelband, etc.)

On 13 November 2017, the Food and Drug Administration (FDA) approved the first pill with a sensor inside of it (aripiprazole tablets with sensor) that can track if a patient has swallowed it. This pill’s sensor sends messages to a wearable patch, and the patch itself transmits the message to a mobile application on the smartphone. This technology could be a game changer for chronic disease and mental health disorders.

**Figure 3:**
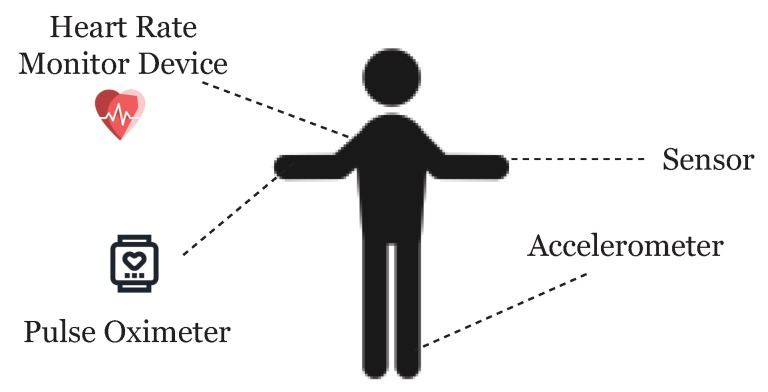
Wearable devices for the patient.

One of the facets of the Internet of Things (IoT) is the network of wearable devices, embedded with software, electronics, sensors, actuators, and connectivity which enables the wearable device to connect and exchange data (Figure 3). In a futuristic smart city, we will not only see these wearable devices transmitting healthcare data, but it is reasonable to assume that wearable devices can share a myriad of data as we interconnect these devices. Therefore, the reach of the ideas presented here regarding wearable healthcare devices and using blockchain technology are further reaching than we show or can imagine here.

To handle such patient data with other institutions, such infrastructure demands secure data sharing. Health data is highly private and sharing of data may raise the risk of exposure. Furthermore, the current system of data sharing uses a centralized architecture which requires centralized trust.

The solution for data privacy and security could and should very well be blockchain technology. Initially proposed by Satoshi Nakamoto in Ref. [1], blockchain technology provides the robustness against failure and data exposure. The blockchain is a shared data structure responsible for storing all transactional history. The blocks relate to each other in the form of a chain. The first block of the chain is known as Genesis. Each block consists of a Block Header, Transaction Counter and Transaction. It acts as a decentralized architecture to record the data. The structure of blockchain is summarized in Table 1.

Blockchain security is based on a *proof of work* concept, and a transaction is only considered valid once the system obtains proof that a enough computational work has been exerted by authorizing nodes. The miners (responsible for creating blocks) constantly try to solve cryptographic puzzles (named Proof of Work (PoW)) in the form of a hash computation. The process of adding a new block to the blockchain is called *mining*. Each block in the chain is identified by a hash in the header. The hash is unique and generated by the Secure Hash Algorithm (SHA-256). SHA takes any size plaintext and calculates a fixed size 256-bit cryptographic hash. Each header contains the address of the previous block in the chain. The inability to delete or change information from blocks makes the blockchain the best appropriate technology for the healthcare system. However, adopting blockchain in the context of IoT is not straightforward and entails several problems such as demands of high computational power to solve PoW, low scalability and long latency for transaction confirmation over the network.

We propose a novel model of blockchain and eliminate the concept of PoW to make it suitable for IoT devices. Our model relies on the distributed nature and other additional security properties to the network. Transactions in blockchain are broadcasted publicly in the blockchain network and contain additional information about both the sender and recipient. If we talk about the blockchain that underpins bitcoin, everyone has a public address, and anyone can see what funds they already have stored at that address. This way, users cannot be anonymous in the network. For anonymity and the authenticity of the user, we present a lightweight privacy-preserving ring signature scheme that is suitable for anonymous transactions by authentic users.

On the one hand, a lightweight digital signature guarantees that information has not been modified, as if it were protected by a tamper-proof seal that is broken if the contents were altered. On the other hand, a ring signature [2] allows a signer to sign a message anonymously. The signature is mixed with other groups (named ring) and no one (except actual signer) knows which member signed the message. We are not using heavy operations such as pairing and exponentiation in ring signature to make it more suitable for blockchain and IoT.

We also use double encryption of data using lightweight encryption algorithms (ARX ciphers) and public encryption schemes. Here double encryption means, we firstly encrypt the data using symmetric key encryption and then we encrypt the symmetric key itself using a public key. Please note that, here we are not encrypting the same data twice with different keys. ARX is a class of cryptographic algorithms which uses three simple arithmetic operations: namely modular addition, bitwise rotation, and exclusive-OR. In both industry and academia, ARX cipher has gained a lot of interest and attention in the last few years. For securely exchanging cryptographic keys over a public channel, we use the Diffie–Hellman key exchange technique. Using both these techniques together will guarantee the security, privacy and anonymity of user’s data using lightweight techniques suitable for small IoT devices.

## 2. Related Work

In this paper, we pull some of our main motivation to explore blockchain in healthcare from Refs. [3,4,5,6,7,8], where the respective authors systematically mentioned some of the latest trends in blockchain research. Since the introduction of Bitcoin in Ref. [1], the possibilities are endless of how the underlying technology can be used in other ways outside of the financial realm. There have been numerous attempts at applying blockchain technology outside of the financial realm [9,10,11,12,13,14]. It is easy to imagine the far-reaching applicability of this technology specifically in healthcare, smart cities and IoT.

In Ref. [15], there was an introduction to methods for using blockchain to provide proof of pre-specified endpoints in clinical trials. Irving and Holden empirically tested such an approach using a clinical trial protocol where outcome switching had previously been reported. They confirmed the use of blockchain as a low cost, independently verifiable method to audit and confirmed the reliability of scientific studies. We use lightweight digital signature schemes in our model inspired by Ref. [2].

We have to date already heard of many data breaches or data losses with regards to medical data [16,17]. Health information is something hackers will seek out as it may contain pertinent information for identity theft. Medical record ownership is another key point when discussing health information. The records themselves come in many forms, reports, images, videos, and raw data. They could potentially also come in many different formats depending on the systems in use by the given provider. The integrity of these records then becomes paramount. There needs to be contingencies in place to ensure the integrity of the data is maintained to ensure the data has not been changed, destroyed, or removed. The access to the data should be controlled by the patients; however, they themselves should not be able to alter it either. The patient records should be consistent and available across institutional boundaries [18,19].

In the context of Smart cities and Big Data, we have seen some strong work recently by Wu and Ota in Refs. [20,21,22,23]. They have really been able to focus on how IoT, Smart Cities, and the resulting Big Data are all important factors going forward with Smart City design and implementation. We have contributed some work already in cryptanalysis of ARX ciphers and other security algorithms [24,25,26,27,28,29,30].

## 3. Drawbacks and Security Issues

The main concern in RPM systems is the secure and efficient transmission of the medical data. Healthcare data is a lucrative target for hackers and therefore securing protected health information (PHI) is the primary motivation of healthcare providers. Healthcare has become the primary target for cybercriminals. For example, cyber-attacks on medical devices or health data have become more common in the last decade. However, the inability to delete or change information from blocks makes blockchain technology the best technology for the healthcare system and could prevent these issues. However, blockchain technology in its original form is not enough of a solution. In this section, we discuss the challenges for applying blockchain to the IoT and explain how to solve these problems in our model.

### System Requirements and Our Solutions

1. **Decentralization:** To ensure robustness and scalability and to eliminate many-to-one traffic flows we need a decentralized system. Using such decentralized systems, we can also eliminate the single point of failure or information delay problems. In our model, we are using an *overlay* decentralized network.
2. **Authentication of data:** User’s computer or cloud services store unpreserved data that needs to be transferred to blockchain networks. During transmission, the data could be modified or lost. The preservation of such incorrect tampered data increases the burden to the system and can cause the loss of the patient (death). Therefore, to ensure that data is not modified, we use a *lightweight digital signature* [2] scheme. On the receiver side, data is verified with the user’s digital signature, and if received correctly, it sends a receipt of data to the patient.
3. **Scalability:** Solving PoW is computationally intensive; however, IoT devices are resource restricted. Also, the IoT network contains many nodes and blockchain scales poorly as the number of nodes in the network increases. We eliminate the concept of PoW in our overlay network and divide our overlay network into several clusters instead of a single chain of blocks, and therefore a single blockchain is not responsible for all nodes. Instead we spread the nodes over several clusters. Our model relies on the distributed nature and other additional security properties to the network.
4. **Data Storage:** Storing IoT big data over blockchain is not practical and therefore we use cloud servers to store encrypted data blocks. The data is safe over the cloud due to additional cryptographic security like the digital signature and high standard encryptions which will be discussed later. However, it may cause a problem about trusted third parties. For this purpose, we store all transactions in different blocks and create a combined hash of each block using Merkle Tree and transfer it to the distributed network. This way, any changes in cloud data can be easily detectable. Doing the storage in this manner also preserves the decentralization over some extents.
5. **Anonymity of users:** Medical data of a patient may contain sensitive information, and therefore data must be anonymized over the network. For anonymity, we are using lightweight Ring structure [2] along with digital signatures. *Ring signature* allow a signer to sign data anonymously, that is the signature is mixed with other groups (named ring), and no one (except actual signer) knows which member signed the message.
6. **Security of data:** Medical devices or health data must be accurate and cannot be changed by hackers. To save the data from hackers, we are using a double encryption scheme. Here double encryption does not refer to encrypting the same data using two keys but instead encryption of the data and again encryption of key which was used to encrypt data. We encrypt the data using lightweight *ARX algorithms* and then encrypt the key using the public key of the receiver. Also, we are using the *Diffie–Hellman key exchange* technique to transfer the public keys and therefore getting the keys is almost impossible for an attacker.

## 4. Our System

Our system consists of five parts: Overlay network, Cloud storage, Healthcare providers, Smart contracts and Patient equipped with healthcare wearable IoT devices.

**Figure 4:**
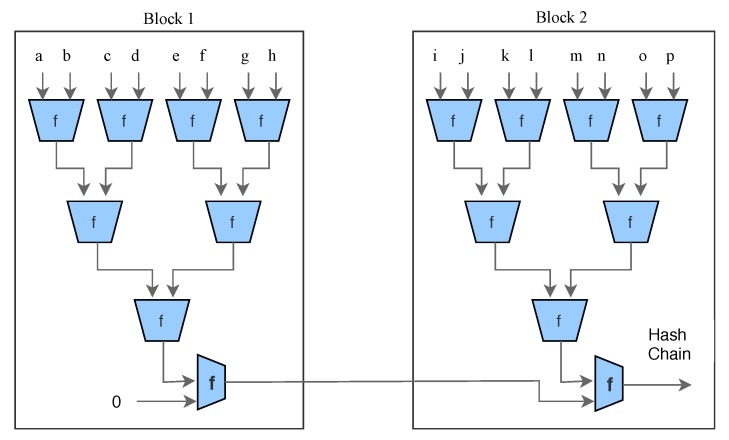
Merkle Tree.

**Figure 5:**
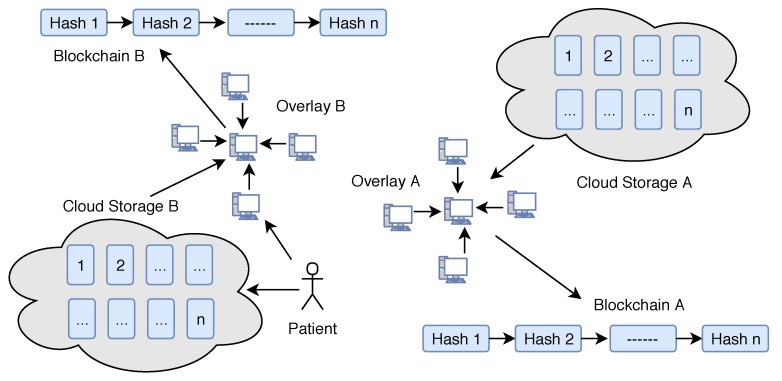
Overlay Network.

1. **Cloud Storage:** Instead of saving the IoT healthcare data over blockchain, we use cloud storage servers to save the patient data. The cloud storage groups user’s data in identical blocks associated with a unique block number. These clouds are connected to overlay networks, once the data stored in a block, the cloud server sends the hash of the data blocks to the overlay network. The hash of the data in the single block is calculated using Merkle Tree (Figure 4). If the overlay network accepts the root hash of the new block, it adds the new hash with the previous hash value and generates the new hash of the chain. In such cases, we do not need any third-party trust, because any changes in data could be easily traceable.
2. **Overlay network:** An overlay is a peer-to-peer network that is based on distributed architecture. The nodes connected to the network could be a computer, smartphone, tablet or any other IoT device as well (Figure 5). (Please note that, in the description of overlay networks we assume that readers have sufficient knowledge of standard cryptographic protocols and use of the hash function in bitcoin mining.) In our model, a network consists of specific nodes and they need to prove that they are certified with a valid certificate. Such a certificate can be uploaded or verified before making an account on the network. Once authorized, he/she will be able to sign data/transaction over the network digitally. To increase network scalability and avoid network delay, we group the nodes in the form of many clusters. Each cluster has one Cluster Head that takes care of public keys of the nodes. Any node attached with any cluster can change the cluster at any time in case of delay. Also, the nodes attached to a cluster can change the cluster head. Cluster head maintains the public keys of requesters (healthcare providers), who can access the data of a particular patient, and the public key of requestees (patient) that are allowed to be accessed.
  Consider the case where a patient wants to share his/her data with a particular doctor, then the node digitally signs the transaction and sends it to the network with a public address of the doctors’ node. The cluster head verifies the patient digital signature and patient public key, and if it is verified correctly, cluster head searches the public key of the doctor node in his own cluster. If the public key is available, then it will broadcast the transaction to its own cluster, and if doctor nodes public key is not available then cluster head will broadcast the transaction to other clusters. In the case where the digital signature or public key of any node is not verified then the cluster will not broadcast data in its cluster but transfer transaction to other cluster heads. Cluster heads are also responsible for storing the hash of the data block stored in the cloud. Each new block in the cluster contains the hash of the previous block also (Please note that each hash block says $hash_{n}$ is combined hash of all previous hashes such as $hash_{1},hash_{2}\ldots hash_{\left(n-1\right)}$). A cluster head can independently decide whether to keep hash of new data block or not. Once a cluster head adds new hash it will broadcast this to all clusters. Other clusters also verify the new block using the hash value of the previous chain. To follow the distributed trust in the network, each cluster head maintains a trust rating for other cluster head based on Beta Reputation System [31]. For more details of overlay networks we suggest readers to reference the following papers [4,32].
3. **Healthcare providers:** Healthcare providers are appointed by insurance companies or by patients to perform medical tests or to provide medical treatments. Healthcare service providers deal with treatment of patients once they receive an alert from the network. They are also treated as a node in the network and authorized to receive particular patient data from the cloud.
4. **Smart contracts:** Smart contracts allow the creation of agreements in any IoT devices which is executed when given conditions are met. Consider we set the condition for the highest and lowest level of patient blood pressure. Once readings are received from the wearable device that do not follow the indicated range, the smart contract will send an alert message to the authorized person or healthcare provider and also store the abnormal data into the cloud so that healthcare providers can receive the patient blood pressure readings as well later on if needed.
5. **Patient with wearable IoT devices:** The IoT device will collect all health data from the patient. Such data could be heartbeats, sleeping conditions, or walking distance to name a few. Patients themselves are the owners of their personal data and responsible for granting, denying or revoking data access from any other parties, such as insurance companies or healthcare providers. If the patient needs medical treatment, he/she will share personal health data with the desired doctor. Once the treatment is finished the patient can deny further access to the doctor, healthcare provider or health insurance company.

## 5. Cryptographic Techniques Used in the Model

**Figure 6:**
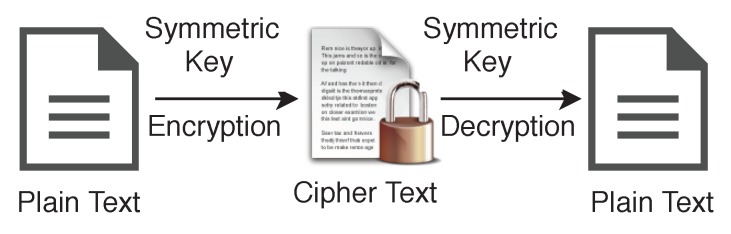
Symmetric Key Encryption.

Instead of only one type of encryption technique, we use both encryption schemes, namely Symmetric and Asymmetric for different purposes. A symmetric algorithm (Private key encryption), as shown in Figure 6, uses the same key for both encryption of plaintext and decryption of ciphertext, whereas asymmetric algorithms (Public key encryption) use different keys for encryption of plaintext and decryption of ciphertext. We use the variable name $k_{sym}$ for the private key or symmetric key in our algorithms, and the same key will be used for encryption and decryption on both side of the transmission.

**Figure 7:**
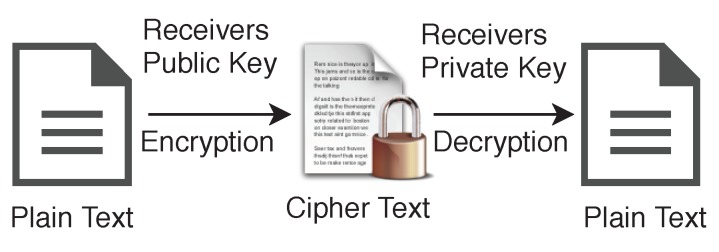
Asymmetric Key Encryption.

In the case of asymmetric encryption, sender will have one key pair $sk_{priv},sk_{pub}$, and receiver will have another key pair $rk_{priv},rk_{pub}$, shown in Figure 7. Data can be encrypted using receiver’s public key $rk_{pub}$ and can be decrypted using their private key $rk_{priv}$. Generally, we use abbreviations plaintext (P) for the unencrypted data and ciphertext (C) for the encrypted data.

### 5.1. ARX Encryption Algorithm

**Figure 8:**
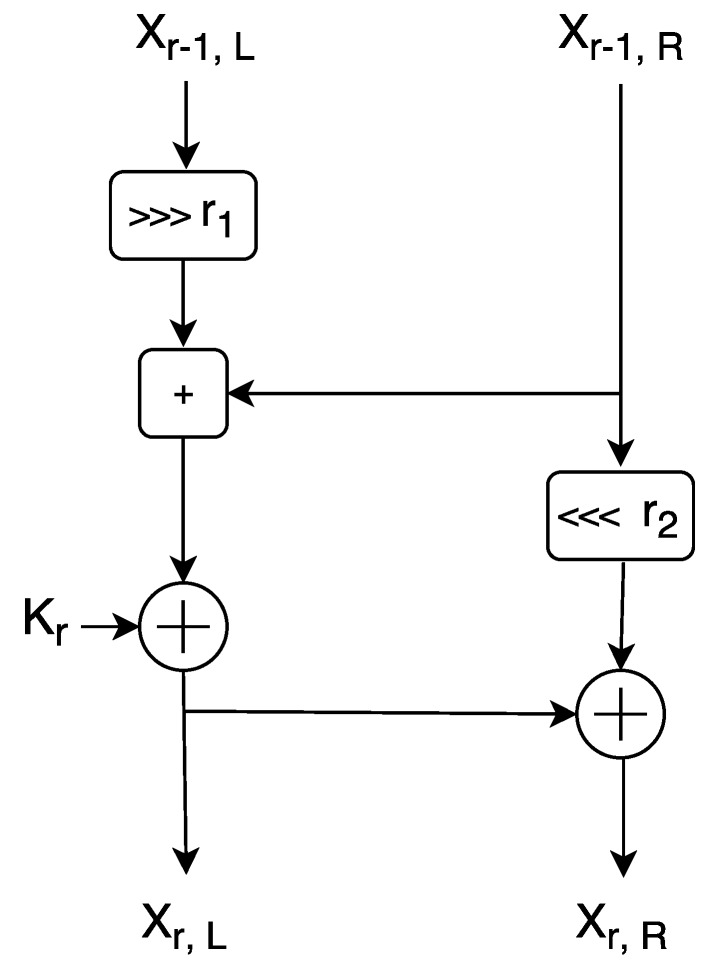
The round function of SPECK.

In our model, we are using a particular branch of Symmetric key encryption, called ARX algorithms to encrypt the data for blockchain. These algorithms are made of simple operations Addition, Rotation and XOR and support a lightweight encryption for small devices. Among a few well known examples, the one example of latest usage of ARX cipher is: SPECK [33], designed by the National Security Agency (NSA), of the United States of America (USA) in June 2013. However, SPECK itself has been severely criticized prior to ISO standardization rejection due to the possibility of the well known cipher backdoor issue, but still, we use it here because it is safe against key recovery attacks. Our model is specifically dedicated to securing the network against various attacks rather than to secure individual nodes. In the case where within the network a defaulter node is found, we can automatically block it. SPECK is a family of lightweight block ciphers with the Feistel-like structure in which each block is divided into two branches, and both branches are modified at every round. We show the round function of SPECK in Figure 8. Each block size is divided into two parts, the left half and right half.

#### SPECK Round Function

SPECK uses 3 basic operations on n-bit word for each round:

- bitwise XOR, ⊕,
- addition modulo $2^{n},⊞$
- left and right circular shifts by $r_{2}$ and $r_{1}$ bits, respectively.

The left half *n*-bit word is denoted by $X_{r-1,L}$ and the right half *n*-bit word is denoted by $X_{r-1,R}$ to the *r*-th round and *n*-bit round key applied in the *r*-th round is denoted by $k_{r}$. $X_{r,L}$ and $X_{r,R}$ denotes output words from round *r* which are computed as follows:

(1) $$X_{r,L}=\left(\left(X_{r-1,L}⋙r_{1}\right)⊞X_{r-1,R}\right)⊕k_{r}$$

(2) $$X_{r,R}=\left(\left(X_{r-1,R}⋘r_{2}\right)⊕X_{r,L}\right)$$

Different key sizes have been used by several instances of the SPECK family and the total number of rounds depends on the key size. The value of rotation constant $r_{1}$ and $r_{2}$ are specified as: $r_{1}=7$, $r_{2}=2$ or $r_{1}=8$, $r_{2}=3$ for various variants of SPECK.

### 5.2. Digital Signature

We add a digital signature to the data for authentication purposes. However, applying normal digital signatures is not suitable due to resources limit in IoT devices. Therefore, we suggest using lightweight digital signatures suitable for small devices as given in Ref. [2]. Digital signatures are the public key primitives of message authentication. Each user has a public-private key pair. Generally, the key pairs used for signing/verifying and the key pairs used for encryption/decryption are different. In our case here, sender will have one key pair $sk_{s_{priv}},sk_{s_{pub}}$, and receiver will have another key pair $rk_{s_{priv}},rk_{s_{pub}}$.

**Figure 9:**
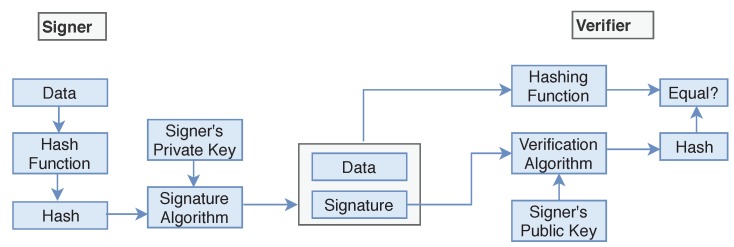
Digital Signature.

The senders private key $sk_{s_{priv}}$ is used to sign the data, and the key is referred to the signature key while senders public key $sk_{s_{pub}}$ is used for verification on the receiver side of the transmission. Signer feeds the data or plaintext into the *Hash Function* and generates the hash value $hash_{p}$. Hash value $hash_{p}$ of plaintext and signature key $sk_{s_{priv}}$ are then fed to the signature algorithm and sent along with the encrypted data (Figure 9). During the verification process, the verifier generates the hash value $hash_{r}$ of the received data from the same hash function. Using the Verification algorithm and signers public key, he/she also extracts the original hash value $hash_{p}$ of plaintext and if the value of $hash_{p}$ and $hash_{r}$ are the same then the data is verified and not changed during the transmission process.

### 5.3. Digital Ring Signature

**Figure 10:**
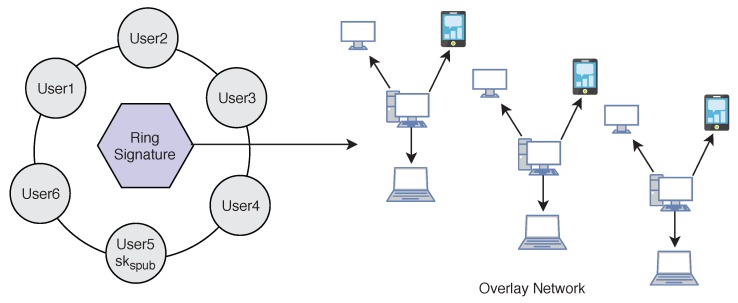
Ring Signature.

We use lightweight *Ring signature* technology [2] which allows a signer to sign data in an anonymous way (Figure 10). That is the signature is mixed with other groups (named ring) and no one (except the actual signer) knows which member signed the message. Ring Signature was originally proposed by Rivest in 2001 [34]. A user desiring to mix his transaction sends a request to the blockchain network. The request comprises the public key $sk_{s_{pub}}$. After receiving the request the network sends back a certain amount of public keys $sk1_{s_{pub}},sk2_{s_{pub}},sk3_{s_{pub}},sk4_{s_{pub}}$ which are collected from other users (u1,u2,…,uN) who also applied for mixing service, including $sk_{s_{pub}}$. Using ring signature in our model we can get two important security properties. We achieve both *Signers Anonymity* and *Signature Correctness*.

1. **Signature Correctness:** A valid signature is always accepted, and an invalid signature is always rejected.
2. **Signers Anonymity:** A signature is produced by one member from the set of public key holders. Therefore, the identity of the signer is always hidden in the network, and no one can find out who is the real signer from the signature.

### 5.4. Diffie–Hellman Key Exchange

In all our previous techniques proposed, we need to transfer the public key through the network. To make data more secure, we also share the public key secretly. To share the public key $sk_{s_{pub}}$ safely along the network we are using the Diffie–Hellman key exchange technique. A basic Diffie–Hellman exchange of a shared secret between Alice and Bob could take place in the following manner:

1. Alice and Bob generate their own private/public keys $\left(k_{A};K_{A}\right)$ and $\left(k_{B};K_{B}\right)$. Both publish or exchange their public keys and keep the private keys for themselves.
2. Clearly, it holds that
  (3) $$S=k_{A}\cdot K_{B}=k_{A}\cdot k_{B}\cdot G=k_{B}\cdot k_{A}\cdot G=k_{B}\cdot K_{A}$$

Alice could privately calculate $S=k_{A}\cdot K_{B}$, and Bob $S=k_{B}\cdot K_{A}$, allowing them to use this single value as a shared secret. For example, if Alice has a message *m* to send Bob, she could hash the shared secret h=H(S), compute x=m+h, and send *x* to Bob. Bob computes $h^{{}^{\prime }}=H\left(S\right)$, calculates $m=x-h^{{}^{\prime }}$, and learns *m*.

An external observer would not be able to easily calculate the shared secret due to the DLP (discrete logarithm problem), which prevents them from ending $k_{A}$ or $k_{B}$. Since the output of hash functions is ‘random’, the message *m* is information-theoretic secure from adversaries who know *x*, $K_{A}$, and $K_{B}$.

## 6. Algorithms to Implement Cryptographic Techniques between Sender and Receiver in Our Model

In our encryption Algorithm 1, we encrypt the data_file by using the symmetric key $k_{sym}$ and produce a ciphertext file *C*. After encryption, we use double encryption technique and encrypt the key $k_{sym}$ by using public key cryptography. We use the receivers public key $rk_{pub}$ to encrypt the symmetric key $k_{sym}$ and send the encrypted key along with the ciphertext *C*. We denote the encrypted symmetric key with $C_{k}$.
**Algorithm 1** Data Encryption.1:**function**Encryption (data_file)2:    **if** user confirm data preservation over blockchain **then**3:        Generate a symmetric key $k_{sym}$4:        $C\leftarrow Encrypt_{sym}$ ($data\_file,k_{sym}$)5:        $C_{k}\leftarrow Encrypt_{asym}\;(k_{sym},rk_{pub}$)6:    **else**7:        Do nothing8:    **end if**9:**end function**

**Figure 11:**
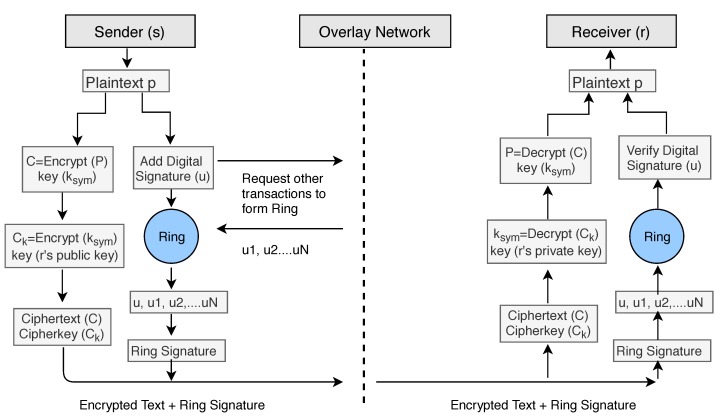
Block Diagram of Model.

For the digital signature senders can use two keys $sk_{s_{pub}},sk_{s_{priv}}$ that is different from the encryption/decryption keys. To add the digital signature, the sender first passes the data file to the *Hash Function* and creates the hash value $hash_{p}$ of the data. Then he/she signs the data using his/her private key $sk_{s_{priv}}$ by passing the value of the private key and hash value $hash_{p}$ to the Signature Algorithm. The signers public key $sk_{s_{pub}}$ can be used to verify data on the receiver’s side. To apply the Anonymity of the patient or user, we add ring signature in our Algorithm 2. The user will ask the network for other accounts who also want to add ring signature to their transactions. The network will provide him/her a set of users who also wish to apply ring signature. The sender’s transaction is then mixed with other users’ transactions and then send it over the network. No one will be able to identify the original signer of the ring group. The process is described in the block diagram (see Figure 11) of our model.
**Algorithm 2** Ring Signature and Public Key Sharing.1:**function**Signature (data_file)2:    **if** user chose anonymity over blockchain **then**3:        Generate an asymmetric public-private key pair $sk_{s_{pub}},sk_{s_{priv}}$4:        $hash_{p}\leftarrow$ calculate hash of the data_file5:        Create the Digital Signature using $hash_{p}$ and signers private key $sk_{s_{priv}}$6:        Share the public key $sk_{s_{pub}}$ to the receiver using Diffie–Hellman key exchange7:        Mix the signature with another network group to form a ring8:    **end if**9:**end function**

To decrypt the ciphertext data *C* (Algorithm 3), we need the symmetric key $k_{sym}$. The symmetric key was encrypted using the public key $rk_{pub}$ of the receiver, and therefore receivers private key $rk_{priv}$ can only decrypt the symmetric key. We firstly decrypt the $C_{k}$ using the private key $rk_{priv}$ of the receiver and get the original symmetric key $k_{sym}$. We apply the key to the ciphertext *C* and get the original plaintext or data file.
**Algorithm 3** Data Decryption.1:**Input:** Encrypted file *C*, Encrypted symmetric key ($C_{k}$)2:**Output:** Decrypted data_file3:**function**Decryption ($C,C_{k},rk_{priv},k_{sym}$)4:    $k_{sym}\leftarrow Decrypt_{asym}\;(C_{k},rk_{priv}$)5:    $data\_file\leftarrow Decrypt_{sym}$ ($C,k_{sym}$)6:**end function**

During the verification process (Algorithm 4), Verifier generates the hash value $hash_{c}$ of received data (ciphertext) using the same hash function. Also, Verifier feeds the digital signature and the verification key into the verification algorithm and extract the hash value $hash_{p}$ of original data (plaintext). If both hash values are equal, it means data file is not modified during transfer between sender and receiver.
**Algorithm 4** Signature Verification.  1:**Input:** Encrypted file *C*, Signers Public key ($sk_{s_{pub}}$)  2:**function**Verification(*C*, $sk_{s_{pub}}$)  3:    $hash_{c}\leftarrow$ calculate hash of the received encrypted data file *C* to be verified  4:    Using Public key $sk_{s_{pub}}$ of signer, extract $hash_{p}$ of senders file  5:    **if** $hash_{c}$ = $hash_{p}$ **then**  6:        return *C*  7:    **else**  8:        return “Signature incorrect”  9:    **end if**10:**end function**

## 7. Model Implementation

In our system, the patient is equipped with wearable devices such as a blood pressure monitor, insulin pump, or other known devices. Random patients are not allowed to connect with the network, they can only connect once they make an account and provide identity verification documents. Once account verifies the documents provided, users are allowed to access the network. The health information is sent to the smart devices such as a smartphone or tablet for the formatting and aggregation by the application (see Figure 1). Once complete, the formatted information is sent to the relevant smart contract for full analysis along with the threshold values as required. The threshold value decides whether the health reading is NORMAL as per standard readings or not. If the health reading is ABNORMAL, then the smart contract will create an event and send an alert to the overlay network and to the patient. Also, it stores the abnormal readings to cloud servers and cloud server can then transfer the hash of the stored data to the overlay network. When health data is transferred to the cloud server, the sender adds a digital signature to the data. Overlay network then sends an alert to the health providers. Here, we are not storing the health readings to the overlay network, but we only store the transaction alert to the overlay network.

**Figure 12:**
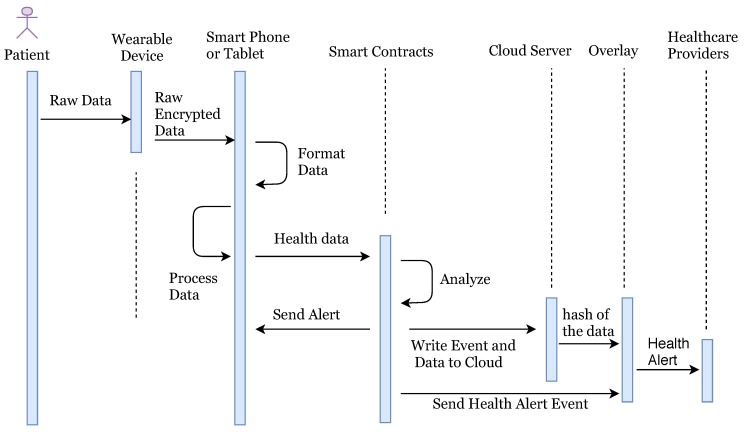
Logical flow execution of the system.

Health Alert Event should also be anonymous, and privacy preserved to the overlay network. We treat this alert as a transaction of the specific user and apply all advanced cryptographic techniques according to the algorithm explained in Section 6. Here the entity who is sending the information could be treated as a sender and who is receiving the information could be treated as a receiver. Here we only describe the flow of data in our system and do not describe all encryption/decryption technical details as we have already explained the cryptographic techniques in above sections by taking a general model of the sender, receiver, and network. An overlay network contains the public key information of all connected nodes and hash index of the stored data over the cloud. Once the healthcare provider node gets an alert, he/she access the full health reading of patient for which he/she is authorized over the network. We summarize the logical flow execution of the system in Figure 12.

## 8. Security Evaluation

In any model, there are three main security requirements that need to be addressed by model designers: Confidentiality, Integrity, and Availability. Confidentiality makes sure that only authorized users can access the system. Integrity is responsible for messages sent to the destination without any change, and Availability means data is always available to the users when needed. We evaluate the security margin of the model under various threats. In this model, the adversary can be a home device, the cluster head, or any other node in the network or part of the cloud storage. These adversaries can discard transactions, sniff communications, create false transactions, or change or delete information from the storage. However, our models’ basic aim is to save the network from the adversary, and we focus on this rather than individual nodes. If a node is connected to the network and he verifies proof of authority and registered by the network, then we assume that he is an honest node. In the case where the network detects some malicious activity by the given node, we can block the malicious node from the network. We summarize the security requirement evaluation in Table 2.

We considered a few attacks that could be possible in this model and find a security margin against them in our model:

1. **Denial of Service (DoS) Attack:** In such an attack, the attacker tries to prevent the authentic user from accessing the service in the network. In such cases, the adversary can launch fraudulent transactions and can increase traffic in the network. However, in our system, random users cannot join the network without proof of authority. Regardless, consider an adversary attacked the network and started sending fake transactions to the network. In such a case, cluster head will check his/her public address and if it is not available or registered with cluster head then it will not broadcast the transaction to the network and forward the request to other clusters. If the public address is not available or is a registered public address, no cluster will accept the request and after many attempts, cluster head will finally block him/her in the network, and therefore our system is safe against this attack. However, a particular attacker can attack many times with a different public address.
2. **Mining Attack:** Consider an adversary hacked a few cluster heads and started controlling multiple cluster heads. In such a situation, fake mining is possible but once it is detected by other cluster heads or nodes, they can easily trace the fake cluster heads. This is because in our model if any cluster head approves a block then it will add a digital signature over that block and without the digital signature other cluster heads will not accept a new block in the network. Once a fake cluster head is detected by the network it can be modified by the nodes in that cluster.
3. **Storage Attack:** If an adversary attacked the cloud storage, he/she can remove, change, or add data in the cloud. However, in our model we are using a hash of the data block stored in the cloud, therefore changes in the data can easily be detected. However, in our model, if any user wants to store or manage data over the cloud, he needs to digitally sign the data and can only access his/her data or others’ data with permission. In such cases, if someone else modifies the data, the cluster heads can block them from the network.
4. **Dropping Attack:** For such an attack, the adversary should have to control cluster heads. The cluster heads under the attacker’s control will not be able to do anything in the network. They will drop all received blocks and they will not be connected to other nodes or clusters. As a solution here the nodes in the network can elect another node as a cluster head.

## 9. Future Work

This paper takes an initial look at a blockchain-based IoT model glimpsing into an advanced security and privacy model to be used in any current IoT-based remote monitoring system. Our main future direction for this work or any researchers who wish to further this work is to implement the system in a testable system to provide some real work security guarantees apart from what has already been established for all the individual cryptographic components used. We also hope to find an industrial partner to help bring some of the novel ideas mentioned in this work to become commercially available.

## 10. Conclusions

IoT privacy and security is one of the most significant issues nowadays in academia and industry. Due to resource constraint factor of IoT, existing security solutions are not well suited. Our proposed architecture provides a solution to most of the security and privacy threats while considering the resource constraint factor of IoT. In this paper, we introduced a novel hybrid approach that combines the advantages of the private key, public key, blockchain and many other lightweight cryptographic primitives to develop a patient-centric access control for electronic medical records, capable of providing security and privacy. We also raise open questions to reduce various attacks such as DoS, modification attacks etc. However, resource-constraints of IoT are key challenges towards answering such problems or seizures.

## Figures and Tables

**Table 1:** Structure of the Blockchain.

Field	Size
Magic Number	4 bytes
Block Size	4 bytes
**Header: Next 80 bytes**	
Version	4 bytes
Previous Block Hash	32 bytes
Merckle Root	32 bytes
Timestamp	4 bytes
Difficulty Target	4 bytes
Nonce	4 bytes
**Rest of Blockchain**	
Transaction Counter	Variable: 1 to 9 bytes
Transaction List	Depends on the transaction size: Upto 1 MB

**Table 2:** Security Requirement Evaluation.

Requirement	Model Solution	Reference
Confidentiality	Proof of Authority, Public Key	Section 6
Authorization	Using Public Key and Lightweight Digital Signature	Section 5.2
User control	Proof of Authority	Section 7
Integrity	Hashing of data blocks	Section 4
Availability	Achieved by limiting acceptable transactions	Section 4
Anonymity	Lightweight Ring Signature	Section 5.3

## Abbreviations

The following abbreviations are used in this manuscript:

*P*	Plaintext or data_file
*C*	Ciphertext or Encrypted data_file
$k_{sym}$	Symmetric Key
$C_{k}$	Encrypted Symmetric Key
$sk_{s_{pub}}$	Senders Public Key for Digital Signature
$sk_{s_{priv}}$	Senders Private Key for Digital Signature
$rk_{s_{pub}}$	Receivers Public Key for Digital Signature
$rk_{s_{priv}}$	Receivers Private Key for Digital Signature
$sk_{pub}$	Senders Public Key for Encryption/Decryption
$sk_{priv}$	Senders Private Key for Encryption/Decryption
$rk_{pub}$	Receivers Public Key for Encryption/Decryption
$rk_{priv}$	Receivers Private Key for Encryption/Decryption
$hash_{c}$	Hash value of the data received at verifier side
$hash_{p}$	Hash value of the plaintext or data send
*u*	user accounts on blockchain
